# Prevalence, Molecular Characterization, and Antibiotic Susceptibility of *Cronobacter sakazakii* Isolates from Powdered Infant Formula Collected from Chinese Retail Markets

**DOI:** 10.3389/fmicb.2017.02026

**Published:** 2017-10-17

**Authors:** Peng Fei, Yichao Jiang, Yan Jiang, Xiujuan Yuan, Tongxiang Yang, Junliang Chen, Ziyuan Wang, Huaibin Kang, Stephen J. Forsythe

**Affiliations:** ^1^College of Food and Biological Engineering, Henan University of Science and Technology, Luoyang, China; ^2^Changbai Mountains Food and Drug Inspection Testing Center, Baishan, China; ^3^Department of Market Supervision and Management, MuLing Food Inspection Testing Center, Mudanjiang, China; ^4^Anda Department of Animal Husbandry and Veterinary, Anda, China; ^5^Foodmicrobe.com, Nottingham, United Kingdom

**Keywords:** *C. sakazakii*, prevalence, genotyping, antibiotic susceptibility, powdered infant formula (PIF)

## Abstract

*Cronobacter sakazakii* is an opportunistic pathogen that causes severe infections in neonates and infants through contaminated powdered infant formula (PIF). Therefore, the aim of this study was a large-scale study on determine the prevalence, molecular characterization and antibiotic susceptibility of *C. sakazakii* isolates from PIF purchased from Chinese retail markets. Two thousand and twenty PIF samples were collected from different institutions. Fifty-six *C. sakazakii* strains were isolated, and identified using *fusA* sequencing analysis, giving a contamination rate of 2.8%. Multilocus sequence typing (MLST) was more discriminatory than other genotyping methods. The *C. sakazakii* isolates were divided into 14 sequence types (STs) by MLST, compared with only seven clusters by *ompA* and *rpoB* sequence analysis, and four *C. sakazakii* serotypes by PCR-based O-antigen serotyping. *C. sakazakii* ST4 (19/56, 33.9%), ST1 (12/56, 21.4%), and ST64 (11/56, 16.1%) were the dominant sequence types isolated. *C. sakazakii* serotype O2 (34/56, 60.7%) was the primary serotype, along with o*mpA*6 and *rpoB*1 as the main allele profiles, respectively. Antibiotic susceptibility testing indicated that all *C. sakazakii* isolates were susceptible to ampicillin-sulbactam, cefotaxime, ciprofloxacin, meropenem, tetracycline, piperacillin-tazobactam, and trimethoprim-sulfamethoxazole. The majority of *C. sakazakii* strains were susceptible to chloramphenicol and gentamicin (87.5 and 92.9%, respectively). In contrast, 55.4% *C. sakazakii* strains were resistant to cephalothin. In conclusion, this large-scale study revealed the prevalence and characteristics of *C. sakazakii* from PIF in Chinese retail markets, demonstrating a potential risk for neonates and infants, and provide a guided to effective control the contamination of *C. sakazakii* in production process.

## Introduction

*Cronobacter* spp. are emerging foodborne opportunistic pathogens that can infect neonates and infants resulting in necrotizing enterocolitis, bacteremia, and meningitis, with a 40–80% mortality rate (Holy and Forsythe, 2014; Li et al., 2016). These organisms have be isolated from various food sources, including spiced meat, ready-to-eat foods, dehydrated rice powder, retail foods, and powdered infant formula (PIF) (Iversen and Forsythe, 2004; Hochel et al., 2012; Joseph et al., 2012a; Huang et al., 2015; Xu et al., 2015; Zhang et al., 2016; Brandão et al., 2017).

The genus *Cronobacter* has been divided into seven species: *Cronobacter sakazakii, Cronobacter malonaticus, Cronobacter turicensis, Cronobacter muytjensii, Cronobacter dublinensis, Cronobacter universalis*, and *Cronobacter condimenti* (Joseph et al., 2012a,b; Yan et al., 2012). Among them, *C. sakazakii* is considered as the predominant species associated with neonatal infections (Forsythe et al., 2014). The consumption of contaminated PIF is the main reason for the occurrence of neonatal infections (Drudy et al., 2006). In production process of PIF, the addition of heat sensitive material, spray drying, fluidized-bed-drying, filling, and packing are the possible links with *C. sakazakii* contamination (Nazarowec-White and Farber, 1997; Pan et al., 2014; Fei et al., 2015). Because of the strong ability to resist desiccation environment, *C. sakazakii* strains can persist in PIF for more than 1 year (Osaili and Forsythe, 2009). Therefore, the presence of *C. sakazakii* in commercial PIF needs to be monitored.

Multilocus sequence typing (MLST), O-antigen serotyping, *ompA* analysis, and *rpoB* analysis can be used to reveal the molecular characterization of *Cronobacter* spp. (Joseph et al., 2012c; Cui et al., 2014; Forsythe et al., 2014; Fei et al., 2015). More than 2,000 *Cronobacter* isolates have been divided into >600 sequence types (STs) using MLST, details of which are recorded in the open access MLST database (http://pubmlst.org/cronobacter/; Forsythe et al., 2014; Ogrodzki and Forsythe, 2017). O-antigen serotyping associated with lipopolysaccharide (LPS) structure is used to type *Cronobacter* strains for epidemiological purposes (Jarvis et al., 2013; Blažková et al., 2015). The O-antigen serotyping scheme based on multiplex polymerase chain reaction (PCR) has been designed, but this method appears to be less discriminatory than MLST which has >600 defined STs (Sun et al., 2012; Mueller et al., 2013; Ogrodzki and Forsythe, 2015). The outer membrane protein A (*ompA*) of *C. sakazakii* plays an important role in invading human intestinal epithelial cells and brain microvascular endothelial cells (Mohan Nair and Venkitanarayanan, 2007; Singamsetty et al., 2008). The sequence analysis of *ompA* gene has been applied to identify and type this pathogen for purposes of pathogenicity (Mohan Nair and Venkitanarayanan, 2006; Fei et al., 2015). Furthermore, *rpoB* allele sequence is also included in the international PubMLST database (Fei et al., 2015). Therefore, a comprehensive comparative analysis of *C. sakazakii* strains isolated from PIF using MLST, O-antigen serotyping, *ompA* scheme, and *rpoB* scheme is warranted.

Currently, antibiotic therapy is the most common and effective method to treat *Cronobacter* infections (Depardieu et al., 2007). A majority of *Cronobacter* spp. stains are reported to be susceptible to frequently-used antibiotics, however, long-term use or abuse of antibiotics is likely to lead to the development of *Cronobacter* antibiotic resistance (Yoneyama and Katsumata, 2006; McMahon et al., 2007). *Cronobacter* strains resistant to amoxicillin-clavulanate, ampicillin, cefazolin, cephalothin, cefotaxime, and streptomycin have been isolated from food samples (Molloy et al., 2009; Ye et al., 2010; Chon et al., 2012; Lee et al., 2012; Pan et al., 2014; Fei et al., 2017). Therefore, it is necessary to evaluate the antibiotic resistance of *Cronobacter* spp. isolated from PIF. PIF is a major food product in China, and the safety of PIF is of particular concern. Our previous study isolated and typed *C. sakazakii* and *C. malonaticus* strains from PIF and production environment of PIF from 2009 to 2012 (Fei et al., 2015). As a continuing research project, the aim of this large-scale study was to determine the prevalence and molecular characterization and of *C. sakazakii* isolates from PIF purchased from Chinese retail markets from January 2015 to March 2017. In addition, the antibiotic susceptibility of these strains was determined to assess any changes in *C. sakazakii* antibiotic resistance compared with earlier studies.

## Materials and methods

### Sample collection

**Figure 1:**
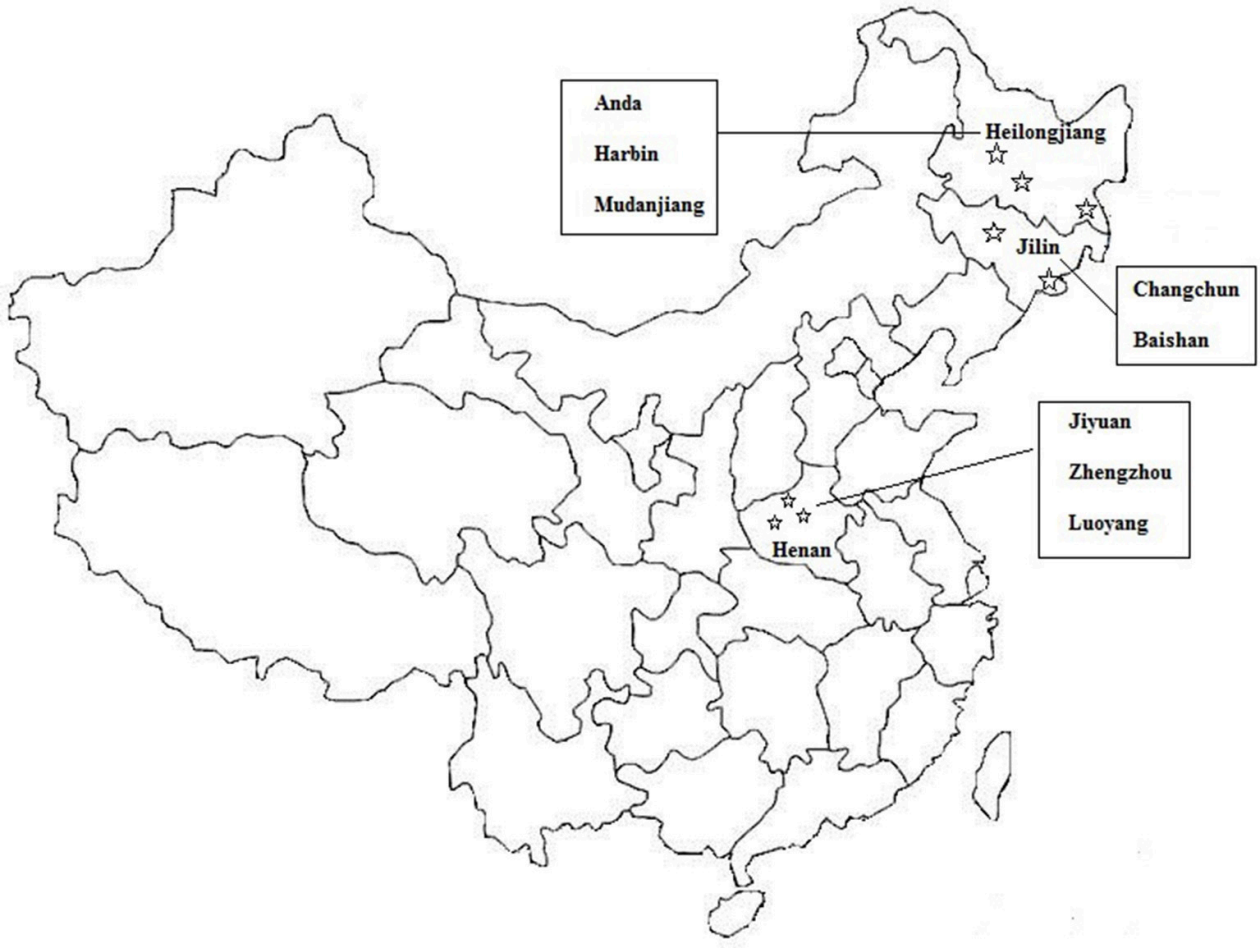
Sampling sites of PIF used in this study.

A total of 2,020 PIF samples were collected from Chinese retail markets for the isolation and identification of *C. sakazakii* strains from January 2015 to March 2017. These PIF samples were from eight cities in three provinces (512 PIF samples from Baishan, 430 PIF samples from Mudanjiang, 300 PIF samples from Harbin, 278 samples from Anda, 200 PIF samples from Changchun, 150 samples from Jiyuan, 100 samples from Luoyang, 50 samples from Zhengzhou; Figure 1, Table S1). Samples were transported to laboratories, and stored cool until further analysis.

### Isolation and identification of bacterial strains

*C. sakazakii* strains were isolated and identified as according to the national food safety standard method for food microbiological examination as used in China GB4789.40-2010 (Ministry of Health of the People's Republic of China, 2010). One hundred gram portions of PIF samples were dissolved in 900 mL of buffered peptone water (BPW, Beijing Obostar Biotechnology Co. Ltd., China), and incubated at 37 ± 1°C for 18 ± 2 h. One milliliter overnight culture was inoculated into 10 mL modified lauryl sulfate tryptose broth-vancomycin medium (mLST-Vm, Beijing Obostar Biotechnology Co. Ltd., China), followed by further selective cultivation at 44 ± 0.5°C or 24 ± 2 h. The cultures were streaked onto Druggan-Forsythe-Iversen (DFI, Beijing Obostar Biotechnology Co. Ltd., China) and incubated at 36 ± 1°C for 24 ± 2 h. Typical *Cronobacter* colonies (blue-green colored colonies) were selected and presumptively identified using the API 20E system. Finally, the identity of the strains was confirmed as *C. sakazakii* using *fusA* sequencing (Joseph et al., 2012c; Forsythe et al., 2014).

### DNA extraction

All isolates were incubated in brain–heart infusion (BHI) broth at 37°C for 18 h, and streaked on Tryptic Soy Agar (TSA) plates, followed by incubation at 37°C for 24 h to obtain isolated colonies. A single colony of each strain was inoculated into the BHI and cultivated at 37°C for 18 h. Approximately 2 mL above-mentioned culture was used to extract genomic DNA of isolates by TIANamp Bacterial DNA Kit (TIANGEN BIOTECH (BEIJING) Co., Ltd., Beijing, China).

### MLST analysis

The MLST scheme was carried out according to Baldwin et al. (2009). Seven housekeeping genes (*atpD, fusA, glnS, gltB, gyrB, infB*, and *ppsA*) was amplified and sequenced in Beijing Genomics Institute (BGI, Beijing China). The sequences were aligned in the *Cronobacter* PubMLST database (http://www.pubmlst.org/cronobacter) to determine type sequence (ST) of *C. sakazakii* isolates. The phylogenetic relationship based on the concatenated sequences composed of seven loci (3,036 bp length) was analyzed using Neighbor-joining algorithm in MEGA6, with 1,000 bootstrap replicates. The equivalent concatenated sequences from *C. sakazakii* ATCC29544^T^, *C. sakazakii* ATCC BAA-894, *C. sakazakii* ATCC29004, *C. sakazakii* ATCC12868, *C. malonaticus* CDC 105877^T^, *C. dublinensis* LMG 23823^T^, *C. turicensis* LMG 23827^T^, *C. universalis* NCTC 9529^T^, *C. condimenti* LMG 26250^T^, and *C. muytjensii* ATCC 51329^T^ were used as species specific reference strains.

### *OmpA* and *rpoB* sequence analysis

The *ompA* and *rpoB* of *C. sakazakii* were amplified as described by previous studies (Mohan Nair and Venkitanarayanan, 2007; Stoop et al., 2009). The PCR products of *ompA* and *rpoB* were sequenced (BGI, Beijing China), and the sequencing results were aligned in *Cronobacter* PubMLST database to determine the allele of *ompA* and *rpoB*.

### O-antigen serotype analysis

*C. sakazakii* isolates were serotyped using multiplex serotyping PCR, mainly according to the previous reports (Jarvis et al., 2011; Sun et al., 2012). Five pairs of primers representing *C. sakazakii* serotypes O1, O2, O3, O4, and O7 were mixed to perform the multiplex serotyping PCR (Sun et al., 2012; Blažková et al., 2015). The sizes of the PCR products were used to determine the serotype of *C. sakazakii* isolates.

### Antibiotic susceptibility testing

The Kirby-Bauer disc diffusion method on the basis of the guidelines of the Clinical Laboratory Standards Institute (CLSI, 2015) was used to evaluate the antibiotic susceptibility of 56 *C. sakazakii* isolates. Ampicillin-sulbactam (10:10 μ g), cephalothin (30 μg), cefotaxime (30 μg), chloramphenicol (30 μg), ciprofloxacin (5 μg), gentamicin (10 μg), meropenem (10 μg), piperacillin-tazobactam (100:10 μg), tetracycline (30 μg), and trimethoprim-sulfamethoxazole (1.25:23.75 μg) were selected for the susceptibility test. The results were expressed as sensitive (S), intermediate (I), and resistant (R) according to the CLSI guidelines. *Escherichia coli* ATCC 25922 was used as the quality control organism.

## Results

### Prevalence of *C. sakazakii* in PIF from Chinese retail markets

*C. sakazakii* strains were isolated from 56 out of 2,020 (2.8%) PIF samples in Chinese retail markets, and were provisionally identified using API 20E system and confirmed using *fusA* sequencing analysis. As shown in Table 1, the highest percentage of *C. sakazakii* isolates was detected in PIF from Anda (3.2%, 9/78), followed by Mudanjiang (3.0%, 13/430), Baishan (2.9%, 15/512), Jiyuan (2.7%, 4/150), Changchun (2.5%, 7/200), Harbin (2.3%, 7/300), Luoyang (2.0%, 2/100), and Zhengzhou (2.0%, 1/50).

**Table 1:** Prevalence and levels of *C. sakazakii* in PIF.

**Bacterial strain**	**Source**	**Region**	**Provider**	**No. of samples**	***C. sakazakii* no. (%)**
CBM1, CBM2, CBM3, CBM4, CBM5, CBM6, CBM7, CBM8, CBM9, CBM10, CBM11, CBM12, CBM13, CBM14, CBM15	PIF	Baishan	A	512	15 (2.9%)
ML1, ML2, ML3, ML4, ML5, ML6, ML7, ML8, ML9, ML10, ML11, ML12, ML13,	PIF	Mudanjiang	B	430	13 (3.0%)
ML14, ML15, ML16, ML17, AD10, AD11, AD12	PIF	Harbin	B&C	300	7 (2.3%)
AD1, AD2, AD3, AD4, AD5, AD6, AD7, AD8, AD9	PIF	Anda	C	278	9 (3.2%)
CBM16, CBM17, CBM18, CBM19, CBM20	PIF	Changchun	A	200	5 (2.5%)
FP1, FP2, FP3, FP4,	PIF	Jiyuan	D	150	4 (2.7%)
FP5, FP6,	PIF	Luoyang	D	100	2 (2.0%)
FP7,	PIF	Zhengzhou	D	50	1 (2.0%)
Total	PIF			2020	56 (2.8%)

*A, Changbai Mountains Food and Drug Inspection Testing Center; B, MuLing Food Inspection Testing Center; C, Anda Department of Animal Husbandry and Veterinary; D, Henan University of Science and Technology*.

### MLST analysis

**Figure 2:**
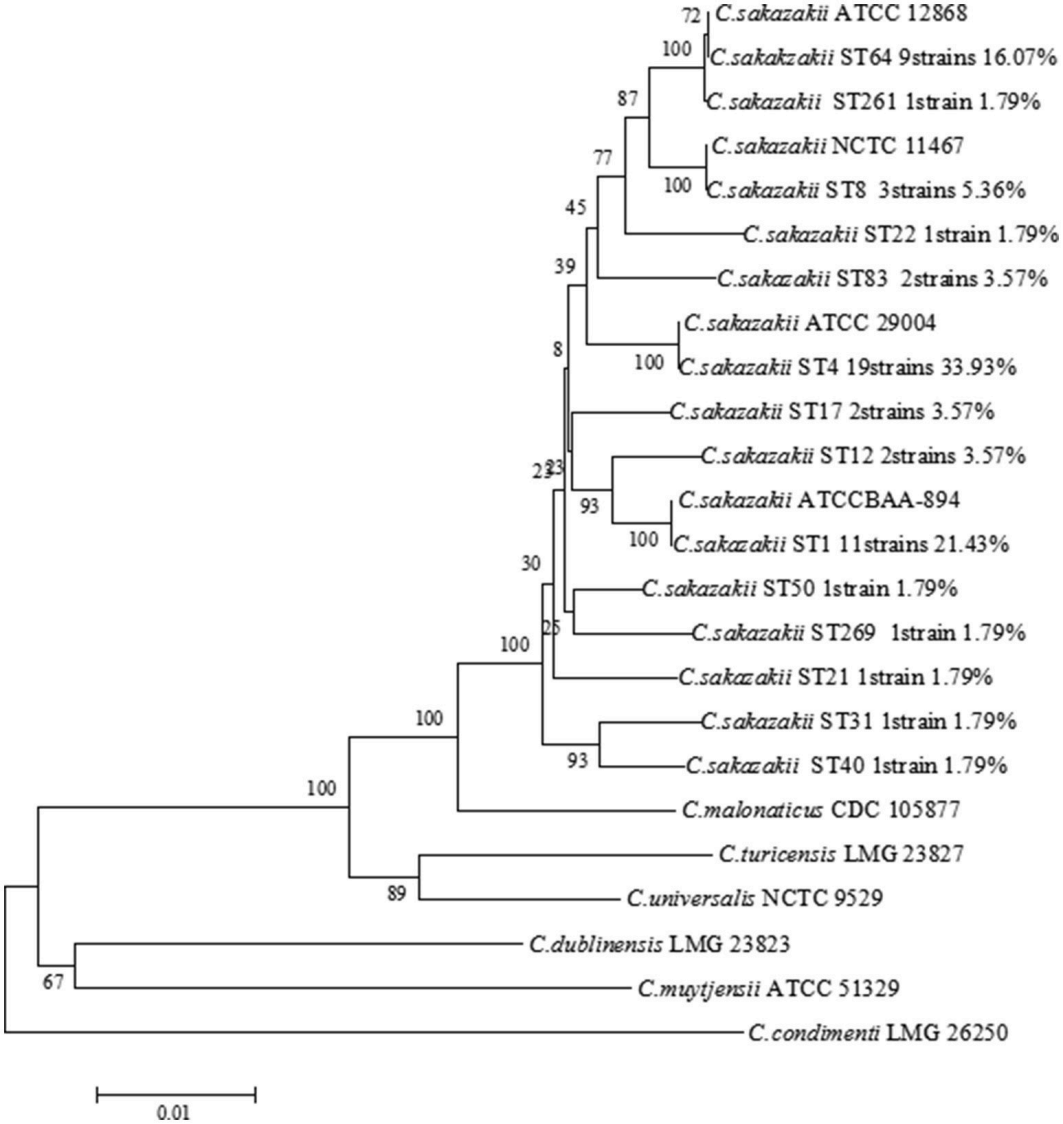
Neighbor-joining tree of MLST 7 loci (3,036 bp) of *C. sakazakii* strains isolated from PIF in Chinese retail markets. *C. sakazakii* ATCC29544^T^, *C. sakazakii* ATCC BAA-894, *C. sakazakii* ATCC29004, *C. sakazakii* ATCC12868, *C. malonaticus* CDC 105877^T^, *C. dublinensis* LMG 23823^T^, *C. turicensis* LMG 23827^T^, *C. universalis* NCTC 9529^T^, *C. condimenti* LMG 26250^T^, and *C. muytjensii* ATCC 51329^T^ were used as the reference strains. The tree was generated using MEGA 6.0 with 1,000 bootstrap replicates.

Fifty-six *C. sakazakii* strains were divided into 14 sequence types, including ST4 (19/56, 33.93%), ST1 (12/56, 21.43%), ST64 (9/56, 16.07%), ST8 (3/56, 5.36%), ST12 (2/56, 3.57%), ST17 (2/56, 3.57%), ST83 (2/56, 3.57%), ST21 (1/56, 1.79%), ST22 (1/56, 1.79%), ST31 (1/56, 1.79%), ST40 (1/56 1.79%), ST50 (1/56, 1.79%), ST259 (1/56, 1.79%), ST261 (1/56, 1.79%), respectively, shown in Table 2. Therefore, ST4, ST1, and ST 64 were considered to be the dominant type sequences of *C. sakazakii* in PIF from Chinese retail markets. The information of all 56 *C. sakazakii* strains were submitted to the *Cronobacter* PubMLST database (http://www.pubmlst.org/cronobacter) with PubMLST IDs 2005 to 2060. A Neighbor-Joining tree based on the concatenated sequences of the seven loci (3,036 bp) for the 56 *C. sakazakii* isolates and 10 reference strains was constructed (Figure 2). The phylogenetic tree showed a clear relatedness between 14 sequence types; ST4, ST1, ST64, ST8, ST12, ST17, ST83, ST21, ST22, ST31, ST40, ST50, ST259, and ST261.

**Table 2:** Molecular characterization of *C. sakazakii* strains isolated from PIF in Chinese retail markets.

**Strain number**	**ID^a^**	**ST^b^**	**CC^c^**	**OT^d^**	***ompA*^e^**	***rpoB*^f^**	**Strain number**	**ID^a^**	**ST^b^**	**CC^c^**	**OT^e^**	***ompA*^f^**	***rpoB*^g^**
CBM1	2005	4	4	O2	6	1	AD12	2033	1	1	O1	3	22
CBM2	2006	4	4	O2	6	1	FP1	2034	1	1	O1	3	22
CBM6	2007	4	4	O2	6	1	FP2	2035	1	1	O1	3	22
CBM10	2008	4	4	O2	6	1	CBM5	2036	64	64	O2	6	35
CBM12	2009	4	4	O2	6	1	CBM8	2037	64	64	O2	6	35
CBM13	2010	4	4	O2	6	1	ML14	2038	64	64	O2	6	35
CBM14	2011	4	4	O2	6	1	ML16	2039	64	64	O2	6	35
CBM15	2012	4	4	O2	6	1	AD6	2040	64	64	O2	6	35
ML1	2013	4	4	O2	6	1	AD7	2041	64	64	O2	6	35
ML2	2014	4	4	O2	6	1	FP4	2042	64	64	O2	6	35
ML4	2015	4	4	O2	6	1	FP6	2043	64	64	O2	6	35
ML5	2016	4	4	O2	6	1	FP7	2044	64	64	O2	6	35
ML10	2017	4	4	O2	6	1	CBM19	2045	8	8	O1	5	21
ML13	2018	4	4	O2	6	1	ML11	2046	8	8	O1	5	21
AD1	2019	4	4	O2	21	1	AD8	2047	8	8	O1	5	21
AD3	2020	4	4	O2	6	1	AD9	2048	12		O4	5	24
AD5	2021	4	4	O2	6	1	AD10	2049	12		O4	5	24
FP3	2022	4	4	O2	6	1	AD11	2050	17	17	O2	6	23
FP5	2023	4	4	O2	6	1	CBM16	2051	17	17	O2	22	23
CBM3	2024	1	1	O1	54	22	CBM20	2052	83	83	O7	6	21
CBM7	2025	1	1	O1	3	22	ML7	2053	83	83	O7	6	21
ML8	2026	1	1	O1	3	22	CBM4	2054	21	21	O1	6	23
CBM9	2027	1	1	O1	3	22	ML9	2055	22		O2	6	1
CBM17	2028	1	1	O1	3	22	ML6	2056	31		O2	23	21
CBM18	2029	1	1	O1	3	22	ML3	2057	40		O4	6	19
ML17	2030	1	1	O1	3	22	ML15	2058	50		O2	21	35
AD2	2031	1	1	O1	3	22	ML12	2059	259		ND	6	1
AD4	2032	1	1	O1	3	22	CBM11	2060	261	64	O2	6	35

a *Strain identification code in the PubMLST Cronobacter database*.

b *ST: Sequence type*.

c *CC: Clonal complex defined as clusters of sequence types with single locus variants*.

d *OT: O-antigen serotype*.

e *Allele numbers of ompA*.

f *Allele numbers of rpoB*.

### *OmpA* and *rpoB* analysis

The nucleotide sequences of *ompA* and *rpoB* were compared with the *Cronobacter* PubMLST database to obtain their allele numbers (Table 2). The 56 *C. sakazakii* strains contained 7 *ompA* allele numbers (*ompA*6, *ompA*3, *ompA*5, *ompA*21, *ompA*22, *ompA*23, and *ompA*54) and 7 *rpoB* allele numbers (*rpoB*1, *rpoB*19, *rpoB2*1, *rpoB*22, *rpoB*23, *rpoB*24, and *rpoB*35), respectively. *OmpA* allele 6 (35/56, 62.5%) was dominant, and included nine sequence types; ST4, ST64, ST17, ST21, ST22, ST40, ST83, ST259, and ST261. Meanwhile, *rpoB* allele 1 (21/56, 37.5%) included three sequence types; ST4, ST22, and ST259 was the main allele number.

### O-antigen serotype analysis

According to the size of the target gene, 56 *C. sakazakii* isolates were divided into several *C. sakazakii* serotypes, including *C. sakazakii* serotype O2 (34/56, 60.71%), *C. sakazakii* serotype O1 (16/56, 28.57%), *C. sakazakii* serotype O4 (3/56, 5.36%), and *C. sakazakii* serotype O7 (2/56, 3.57%; Table 2). The *C. sakazakii* serotype O2 was the dominant serotype for PIF from Chinese retail markets, and was composed of *C. sakazakii* ST4, ST64, ST17, ST22, ST31, ST50, and ST261. In addition, *C. sakazakii* serotype O1 included *C. sakazakii* ST1, ST8, ST21, *C. sakazakii* serotype O4 was composed of *C. sakazakii* ST12 and ST40, *C. sakazakii* serotype O7 contained two strains which belonged to ST83. The serotype of *C. sakazakii* ML12 (ST259) could not be determined using the standard multiplex serotyping PCR method.

### Antibiotic resistance profiles

The antibiotic susceptibility of the 56 *C. sakazakii* strains isolated from PIF is shown in Table 3. All *C. sakazakii* isolates were susceptible to ampicillin-sulbactam, cefotaxime, ciprofloxacin, meropenem, tetracycline, piperacillin-tazobactam, and trimethoprim-sulfamethoxazole. The majority of *C. sakazakii* strains were susceptible to chloramphenicol and gentamicin, with sensitive rates of 87.5 and 92.9%, respectively. In contrast, most *C. sakazakii* strains were resistant to cephalothin, with resistance and intermediate rates of 55.4 and 41.0%, respectively.

**Table 3:** Antibiotic susceptibility of 56 *C. sakazakii* strains isolated from PIF in Chinese retail markets.

**Antimicrobial agent**	***C. sakazakii* strains (*n* = 56)**		
	**No. (%) of R**	**No. (%) of I**	**No. (%) of S**
Ampicillin-sulbactam, cefotaxime, ciprofloxacin, meropenem, Piperacillin-tazobactam, tetracycline, trimethoprim-sulfamethoxazole	0 (0.0)	0 (0.0)	56 (100.0)
Cephalothin	31 (55.4)	23 (41.0)	2 (3.6)
Chloramphenicol	5 (8.9)	2 (3.6)	49 (87.5)
Gentamicin	2 (3.6)	2 (3.6)	52 (92.9)

## Discussion

*C. sakazakii* is the dominant species in *Cronobacter* spp. associated with the infection of newborns through contaminated PIF, therefore, the issue of PIF contamination by *C. sakazakii* is a matter of continuing concern. Many studies have focused on the isolation and identification of *Cronobacter* spp. in PIF for evaluating the contamination of PIF by *C. sakazakii* and related species (FAO/WHO, 2004, 2008; Hoque et al., 2010; Pan et al., 2014; Xu et al., 2014). In our previous study, 66 *C. sakazakii* strains and 4 *C. malonaticus* strains were isolated from 1,228 PIF samples and a wet processing factory of PIF between 2009 to 2012 (Fei et al., 2015). As a continuing study, 56 *C. sakazakii* strains were isolated and identified from 2,020 PIF samples from Chinese retail markets sampled between July 2015 and March 2017. Giving a contamination rate of 2.8%. The contamination rate in this study is lower than the previous data provided by Pan et al. (2014) (12.3%, 49 out of 399) and Xu et al. (2014) (4.3%, 23 out of 530). Our results can contribute toward to an improved understanding and improvement in the surveillance of *C. sakazakii* in commercial PIF available in China.

The samples used for this test were collected from eight cities in three provinces. In the three provinces, the main sequence types of isolates from PIF in retail markets were ST4, ST1, and ST64, which agrees with previous studies and two of which (ST1 and ST4) are major *Cronobacter* pathovars (Sonbol et al., 2013; Fei et al., 2015; Ogrodzki and Forsythe, 2017). However, there were some difference in the composition of STs between three provinces. A total of 12 *C. sakazakii* STs were found in PIF from Heilongjiang province, among them, ST12, ST22, ST31, ST40, ST50, and ST259 were not detected in both Jilin province and Henan province. Eight *C. sakazakii* STs were isolated from PIF collected from Jinlin province, ST21 and ST261 were unique in this region. In Henan province, only three *C. sakazakii* STs (ST4, ST1, and ST64) were found. These finding revealed the relationship between *C. sakazakii* STs and regions, which contribute to make better targeted prevention and control measures in the different regions.

A total of 56 *C. sakazakii* isolates were genotyped into 14 STs by MLST, among them, *C. sakazakii* ST4 was the main sequence type of *Cronobacter* spp., and was associated with neonatal meningitis (Joseph and Forsythe, 2011; Joseph et al., 2012c; Forsythe et al., 2014). Meanwhile, *C. sakazakii* isolates belonging to ST4 had a stronger ability to resistance to desiccation than ST1, ST8, ST12, ST21, ST64, ST201, and ST258, which may be one of reasons that ST4 was the main sequence type recovered from PIF (Fei et al., 2017). *C. sakazakii* ST83 is another major sequence type with a strong capacity to resistance to desiccation in PIF factories (Chase et al., 2017). *C. sakazakii* ST1 is reported to be a major sequence type of strains from PIF, while *C. sakazakii* ST8 strains are primarily isolated from clinical sources (Sonbol et al., 2013). In addition, *C. sakazakii* ST12 can infect neonates and infants to suffer from necrotizing enterocolitis (Forsythe et al., 2014). The *C. sakazakii* strains with these STs have been isolated from commercial PIF, which suggests that ST4, ST1, ST8, ST12, and ST83 should be more risk for neonates and infants.

*OmpA* and *rpoB* analysis can be used to identify and genotype the *Cronobacter* spp. *OmpA*6 was the main cluster of *C. sakazakii* isolated from PIF in Chinese retail markets, and corresponded with *C. sakazakii* ST4 associated with neonatal meningitis, besides, *ompA*21 also been found in *C. sakazakii* ST4 strains. Meanwhile, *rpoB*1 containing ST4, ST22, and ST259 was the predominant, and overlapped with those in ompA6. In addition, compared with MLST, the *ompA* and *rpoB* analysis were less discriminatory.

O-antigen serotype analysis can improve the understanding of *C. sakazakii* on pathogenicity. Previously, *C. sakazakii* species had been classified into seven O-antigen serotypes (Sun et al., 2012). However, a new report indicated *C. sakazakii* serotype O5 and O6 should be classified as *C. malonaticus* serotype O2 and O3, respectively (Blažková et al., 2015). Therefore, in this study, five pairs of primers representing *C. sakazakii* serotypes O1, O2, O3, O4, and O7 were mixed to perform the multiplex serotyping PCR. Meanwhile, *C. sakazakii* serotype O2 and O1 were the main O-antigen serotypes, which had been confirmed to be particularly predominant in clinical cases by Blažková et al. (2015). *C. sakazakii* ST83 and *C. sakazakii* O7 strains can survive in PIF and PIF processing environment for several years, and infect neonates with a high risk (Chase et al., 2017). Our result indicated there was a correlation between O-antigen serotype O7 and ST 83, which was consistent with the finding of Mueller et al. (2013).

Antibiotic susceptibility tests showed that all 56 *C. sakazakii* strains were susceptible to ampicillin-sulbactam, cefotaxime, ciprofloxacin, meropenem, piperacillin-tazobactam, tetracycline, and trimethoprim-sulfamethoxazole. Similarly, the resistance of these antibiotics in *Cronobacter* spp. isolates from PIF, ready-to-eat foods, Brazilian retail foods, and desiccated foods in Korea is common (Chon et al., 2012; Hochel et al., 2012; Xu et al., 2015; Fei et al., 2017). In addition, 8.9 and 3.6% isolates were resistant to chloramphenicol and gentamicin, respectively. This ratio was greater than previous reports (Al-Nabulsi et al., 2011; Lee et al., 2012; Zhang et al., 2016), which may be due to the continued use of antibiotics in clinical practice (Yoneyama and Katsumata, 2006).

In conclusion, the contamination of *C. sakazakii* strains in PIF was still evident in products from Chinese retail markets. The finding of our study detected the prevalence and levels of *C. sakazakii* strains in PIF from Chinese retail markets, and revealed the molecular characterization and antibiotic resistance of these isolates. These results contributes to monitoring the contamination of commercial PIF for *C. sakazakii*, and provide a basis for improved control and reduce neonatal exposure to the organism.

## Author contributions

Conceived and designed the experiments: PF, HK, and SF. Performed the experiments: PF, YiJ, YaJ, XY, and ZW. Generated and analyzed the data: TY and JC. Wrote the paper: PF, HK, and SF.

### Conflict of interest statement

The authors declare that the research was conducted in the absence of any commercial or financial relationships that could be construed as a potential conflict of interest.
