# Supplementary material for: Prevalence, Molecular Characterization, and Antibiotic Susceptibility of Cronobacter sakazakii Isolates from Powdered Infant Formula Collected from Chinese Retail Markets
Source: Front Microbiol. 2017 Oct 17;8:2026. doi: 10.3389/fmicb.2017.02026 (PMC5651101; doi:10.3389/fmicb.2017.02026)
Supplement: Supplementary file 1 [file Table1.DOC]

S Table1 The detailed information of samples used in this study. A-H represented different manufacturers

| No. of samples | [region](../../../../C:/Users/Administrator/AppData/Local/youdao/Dict/Application/7.2.0.0703/resultui/dict/%3Fkeyword=region) | manufacturers | Date of production | Date of collection |
| --- | --- | --- | --- | --- |
| 25 | Baishan | A | 06/01/2015 | 17/02/2015 |
| 25 | Baishan | B | 24/10/2014 | 17/02/2015 |
| 25 | Baishan | C | 07/02/2015 | 17/02/2015 |
| 25 | Baishan | D | 26/11/2014 | 17/02/2015 |
| 25 | Baishan | E | 10/01/2015 | 17/02/2015 |
| 50 | Baishan | A | 17/02/2015 | 20/10/2015 |
| 25 | Baishan | B | 24/04/2015 | 20/10/2015 |
| 25 | Baishan | C | 10/10/2015 | 20/10/2015 |
| 25 | Baishan | D | 10/04/2015 | 20/10/2015 |
| 25 | Baishan | E | 16/08/2015 | 20/10/2015 |
| 30 | Baishan | A | 09/03/2016 | 13/05/2016 |
| 20 | Baishan | B | 27/04/2016 | 13/05/2016 |
| 25 | Baishan | C | 20/02/2016 | 13/05/2016 |
| 25 | Baishan | D | 10/04/2016 | 13/05/2016 |
| 25 | Baishan | E | 16/03/2016 | 13/05/2016 |
| 25 | Baishan | A | 08/06/2016 | 30/11/2016 |
| 25 | Baishan | B | 17/05/2016 | 30/11/2016 |
| 25 | Baishan | C | 21/06/2016 | 30/11/2016 |
| 25 | Baishan | D | 09/09/2016 | 30/11/2016 |
| 12 | Baishan | E | 11/10/2016 | 30/11/2016 |
| 20 | Mudanjiang | A | 19/03/2015 | 20/05/2015 |
| 20 | Mudanjiang | B | 27/02/2015 | 20/05/2015 |
| 20 | Mudanjiang | C | 06/03/2015 | 20/05/2015 |
| 20 | Mudanjiang | F | 19/04/2015 | 20/05/2015 |
| 20 | Mudanjiang | G | 14/03/2015 | 20/05/2015 |
| 20 | Mudanjiang | A | 11/09/2015 | 12/12/2015 |
| 20 | Mudanjiang | B | 03/09/2015 | 12/12/2015 |
| 20 | Mudanjiang | C | 13/10/2015 | 12/12/2015 |
| 20 | Mudanjiang | F | 28/08/2015 | 12/12/2015 |
| 20 | Mudanjiang | G | 22/09/2015 | 12/12/2015 |
| 20 | Mudanjiang | A | 17/01/2016 | 23/06/2016 |
| 20 | Mudanjiang | B | 20/02/2016 | 23/06/2016 |
| 20 | Mudanjiang | C | 14/04/2016 | 23/06/2016 |
| 20 | Mudanjiang | F | 27/03/2016 | 23/06/2016 |
| 20 | Mudanjiang | G | 12/02/2016 | 23/06/2016 |
| 20 | Mudanjiang | A | 11/12/2016 | 01/03/2017 |
| 20 | Mudanjiang | B | 28/12/2016 | 01/03/2017 |
| 20 | Mudanjiang | C | 01/02/2017 | 01/03/2017 |
| 20 | Mudanjiang | F | 24/12/2016 | 01/03/2017 |
| 20 | Mudanjiang | G | 26/01/2017 | 01/03/2017 |
| 30 | Mudanjiang | D | 17/12/2017 | 01/03/2017 |
| 50 | Harbin | A | 29/04/2015 | 18/07/2015 |
| 50 | Harbin | B | 11/03/2015 | 18/07/2015 |
| 50 | Harbin | C | 16/05/2015 | 18/07/2015 |
| 50 | Harbin | A | 14/06/2016 | 20/09/2016 |
| 50 | Harbin | B | 11/04/2015 | 20/09/2015 |
| 50 | Harbin | C | 28/05/2015 | 20/09/2015 |
| 25 | Anda | A | 15/01/2015 | 08/03/2015 |
| 25 | Anda | B | 02/12/2014 | 08/03/2015 |
| 25 | Anda | D | 28/02/2015 | 08/03/2015 |
| 25 | Anda | F | 28/11/2014 | 08/03/2015 |
| 25 | Anda | A | 19/12/2015 | 09/03/2016 |
| 25 | Anda | B | 04/02/2016 | 09/03/2016 |
| 25 | Anda | D | 13/01/2016 | 09/03/2016 |
| 25 | Anda | F | 28/08/2015 | 09/03/2016 |
| 25 | Anda | A | 20/11/2016 | 06/03/2017 |
| 25 | Anda | B | 16/02/2017 | 06/03/2017 |
| 25 | Anda | D | 12/12/2016 | 06/03/2017 |
| 25 | Anda | F | 19/01/2017 | 06/03/2017 |
| 33 | Changchun | A | 13/04/2016 | 23/09/2015 |
| 33 | Changchun | C | 07/03/2016 | 23/09/2015 |
| 34 | Changchun | E | 22/08/2016 | 23/09/2015 |
| 36 | Changchun | A | 18/03/2016 | 04/07/2016 |
| 30 | Changchun | C | 19/05/2016 | 04/07/2016 |
| 34 | Changchun | E | 15/04/2016 | 04/07/2016 |
| 25 | Jiyuan | A | 27/08/2016 | 19/11/2016 |
| 25 | Jiyuan | G | 16/09/2017 | 19/11/2016 |
| 25 | Jiyuan | H | 09/07/2016 | 19/11/2016 |
| 25 | Jiyuan | A | 21/12/2016 | 17/02/2017 |
| 25 | Jiyuan | G | 25/01/2017 | 17/02/2017 |
| 25 | Jiyuan | H | 08/11/2016 | 17/02/2017 |
| 25 | Luoyang | A | 06/01/2017 | 26/02/2017 |
| 25 | Luoyang | C | 30/11/2016 | 26/02/2017 |
| 25 | Luoyang | G | 22/12/2016 | 26/02/2017 |
| 25 | Luoyang | H | 13/01/2017 | 26/02/2017 |
| 10 | Zhengzhou | A | 06/01/2017 | 12/03/2017 |
| 10 | Zhengzhou | B | 30/01/2017 | 12/03/2017 |
| 10 | Zhengzhou | C | 16/02/2017 | 12/03/2017 |
| 10 | Zhengzhou | H | 27/12/2016 | 12/03/2017 |
| 10 | Zhengzhou | G | 21/02/2017 | 12/03/2017 |
